# Peer Review of “COVID-19 National Football League (NFL) Injury Analysis: Follow-Up Study”

**DOI:** 10.2196/55997

**Published:** 2024-02-13

**Authors:** Ari Samaranayaka

**Affiliations:** 1 University of Otago, Dunedin, New Zealand

**Keywords:** COVID-19, injury, prevalence, adaptation, sports medicine, follow-up, training, football, epidemiology, sport, athlete, athletic, injuries

*This is the peer-review report for “COVID-19 National Football League (NFL) Injury Analysis: Follow-Up Study.”*

## Round 1 Review

### General Comments

#### My Review—COVID-19 NFL Injury Prevalence Analysis, A Follow-Up Study

Throughout the manuscript [1], including in the title, the authors say they analyzed the prevalence of injuries. This is incorrect. They have not analyzed injury prevalence; they did not even collect the data required for such an analysis. Instead, they collected injury incidence data and analyzed them.

The primary component of this study is analyzing publicly available data to make a conclusion. I have a major concern regarding the statistical analysis the authors have performed. They have collected injury incidence data for each week for each team over the season from publicly available sources. This includes injuries from the same team for each week, which is repeated data. They then calculated the mean per week per team. They had 32 teams and therefore have 32 means for a season. They then compared the mean of those means between seasons using an unpaired *t* test. First, this analysis totally ignores complications due to nonindependence in repeated data. Second, how can we understand the comparison of the means of means? Third, they compared each possible pairs of years. They ignored the multiple comparison issue. This analysis is totally inappropriate. I am not going to accept the results of this analysis, or any conclusion based on these results. This is an issue that cannot be rescued by a revision.

The authors say they have done a similar analysis in their precious paper [2]. I now doubt the findings published there too. Unfortunately, that paper was also published in *JMIR*. I recommend that editors should consider rereviewing that paper by an independent statistical reviewer.

There are less severe issues as well. For example, they presented 2 figures—one is redundant in the presence of the other, because the numbers in Figure 1 divided by the number of weeks are the numbers in Figure 2. Further, none of the numbers in any of these figures are the outcome measure they used in the statistical analysis. Therefore, the usefulness of them is limited only to describing the raw data.

Even if the analysis is correct, they have a fundamental limitation in their interpretation of the results. Their conclusions are based on the underlying assumption that the observed statistical differences were driven by training opportunities. There was no justification for that assumption. How can the authors claim none of the other possible influencing factors changed?

## References

[1] Puga TB, Schafer J, Thiel G (2024). COVID-19 National Football League (NFL) injury analysis: follow-up study. JMIRx Med.

[2] Puga TB, Schafer J, Agbedanu PN, Treffer K (2022). COVID-19 return to sport: NFL injury prevalence analysis. JMIRx Med.

